# Rhus Aromatica in the Treatment of Incontinence of Urine

**Published:** 1891-10

**Authors:** William C. Krauss

**Affiliations:** Professor of Pathology in the Medical Department of Niagara University, Buffalo, N.Y.; 382 Virginia Street


### RHUS AROMATICA IN THE TREATMENT OF INCONTINENCE OF URINE.

By WILLIAM C. KRAUSS, M. D.,

Professor of Pathology in the Medical Department of Niagara University, Buffalo, N. Y.

Rhus aromatica is extracted from the bark of the root of the sweet sumach, natural order anacardiaceæ, and contains a resin, volatile oil, and tannin. Shoemaker gives its physiological action as astringent, tonic, and diuretic.

Within the past two years much has been said and written of its adaptability to diseases of the genito-urinary tract, and particularly to incontinentia urinæ, enuresis. Many contributions have already appeared, among others by Max, Burvenich, Numa, Hanon, Eloy, and Powell, and all speak highly of its curative, perhaps specific, qualities in the treatment of one of the most obstinate, as well as disagreeable of infantile affections. Having been attracted by these glowing results, and having some cases at hand, I determined to test its virtues and verify for myself the praise so unstintingly bestowed upon it.

For convenience sake we may divide the etiology of enuresis into four groups.

The first group comprises slight functional disturbances of the genito-urinary tract, and produce incontinence by maintaining irritation, which causes an undue sensitiveness of the mucous lining of the bladder. As such may be mentioned, a tightened or adherent prepuce, a narrow meatus, a sensitive clitoris, a sensitive urethra, weakness of the sphincter, cystitis, hyperacidulated urine, pressure on the bladder during pregnancy, obstipation, the presence of ascarides in the rectum, etc.

Disorders of the central nervous system very often produce incontinence in children. Precocious development or defective mental development, dreams, extreme activity of the nerve centers producing disordered sleep, and the continuance of the habit which was set up in infancy, are some of the most important of this class.

Under the third head we may group those organic diseases of the spinal cord which engender incontinence by destroying the vesical center, or by injuring the reflex arc. Diseases affecting the posterior white columns, as locomotor ataxia, transverse myelitis, spinal tumors, etc., belong to this group.

The fourth group embraces organic changes along the genito-urinary system, more especially of the bladder and its appendages. Hypertrophy of the muscular coat of the bladder as met with in women and elderly men, enlargement of the prostate, and atony of the bladder walls leading to retention and later on incontinence, are examples of this kind.

The treatment which I have formulated for these different groups is, briefly, as follows:

1st. Remove the cause of irritation, if possible, by operation or necessary treatment, and administer the fluid extract of rhus aromatica, beginning with five to ten drops, and increase to fifteen–twenty, four times daily. I generally prescribe it with glycerine, to be taken after meals and before retiring.

2d. A general nerve tonic or nerve sedative treatment auxiliary with the rhus will prove of great benefit. In anemic cases I combine the rhus with the syrup of the iodide of iron, as

℞—Rhus aromatica, fl. ext. . . . . . . . . . . . . . . . . ʒ v.
Syr. ferri iodidi . . . . . . . . . . . . . . . . . . . . . .
Elixir calisaya . . . . . . . . . . . . . . . . . . . . . . . āā q. s. ad ℥ ii.
M. Sig.—One-half teaspoonful four times daily.

Incontinence of urine due to any of the causes in groups one and two yields readily to the administration of rhus aromatica.

3d. In these cases, especially in adults, where there exists organic disease of the spinal cord destroying the vesical center or its arc, rhus aromatica has had no effect whatever. I have used it in combination with the fluid extract of ergot, of each twenty drops, but have as yet been unable to discover any favorable results.

4th. Under the last head, where there is more or less hypertrophic paralysis of the vesical apparatus, I would prescribe rhus in half-drachm doses, supplemented by local treatment. I have had no experience with this group, and cannot speak knowingly.

A brief summary of my cases, with results, is here appended:

Case I.—Female, age, 28; has been troubled with incontinence for years, all manner of treatment having heretofore been unsuccessful. The patient is of a neurotic temperament, with symptoms of neurasthenia. I prescribed rhus aromatica, twenty drops four times daily, along with a general treatment, and at the end of four weeks she considered herself cured of the incontinence. One year has now elapsed without any recurrence of the trouble.

Case II.—Female, age, 14; has had nocturnal incontinence since childhood. She is anemic, of a nervous temperament, and presents some symptoms of exophthalmic goitre. I prescribed rhus with syrup of iron, as per formula, and at the end of five weeks the enuresis disappeared.

Case III.—Male, age, 13; has had incontinence for the past four years. An examination revealed an adherent prepuce with accumulations of smegma. Removing the local cause and administering rhus in twenty-drop doses, along with the syrup of iron, cured the incontinence in two weeks' time.

Case IV.—Female, age, 29; in the third month of pregnancy. For the past four weeks has complained of involuntary urination. I prescribed rhus with glycerine, and at the end of two weeks she complained of no further trouble.

Case V.—Female, age, 6; of a highly nervous temperament; has had incontinence, nocturnal and diurnal, since birth. As a result of the long continued irritation she has had, at times, epileptic manifestations. After a thorough treatment with bromides and rhus, both epilepsy and incontinence disappeared.

Case VI.—Female, age, 70; presents paraplegia with incontinence of urine and feces, and slight sensory disturbances, symptoms indicating a transverse myelitis. Rhus and ergot, in proportions above given, after three months' trial, were discontinued without any results.

Case VII.—Female, age, 45; and

Case VIII.—Male, age, 38; both suffering with symptoms of locomotor ataxia, failed to receive any benefit from the rhus treatment.

Case IX.—Female, age, 65; has been troubled for some weeks with a girdle pain starting from the upper lumbar region, accompanied with paraplegia, incontinence of urine, and feces, and slight disorders of sensation. The patellar reflexes at first were exaggerated, but later disappeared entirely. The onset and course of the malady points strongly to a spinal tumor in the upper lumbar region. Rhus aromatica has been given for some time without affording any relief, and was consequently suspended.

Besides the internal use of rhus aromatica, I generally advise my patients to bear in mind the following rules: Bathe the parts with cold water just before retiring. Urinate during the day as soon as a desire is present, and urinate before retiring. Eat a light lunch, and avoid all liquid foods during the evening. Enjoy good air and good morals; avoid a strict meat diet, manipulation of the parts, and undue excitement.

### CONCLUSIONS.

Incontinentia urinæ due to slight disorders of the genito-urinary or nervous systems is amenable to the rhus treatment, and gives most favorable results.

Incontinentia urinæ due to destructive lesions of the spinal cord complicating the vesical center or its reflex arc, is not amenable to the rhus treatment, and gives negative results.

382 Virginia Street.

